# Role of Suppressors of Cytokine Signaling 3 in Bone Inflammatory Responses

**DOI:** 10.3389/fimmu.2013.00506

**Published:** 2014-01-10

**Authors:** Anqi Gao, Thomas E. Van Dyke

**Affiliations:** ^1^Department of Applied Oral Sciences, The Forsyth Institute, Cambridge, MA, USA

**Keywords:** SOCS3, bone, inflammation

## Abstract

Suppressor of cytokine signaling 3 (SOCS3) is a potent regulator of cytokine signaling in macrophages and T cells. In recent studies, evidence has been provided for SOCS3 activation in all major bone cells including osteoclasts, chondrocytes, synoviocytes, and osteoblasts. The investigation of SOCS3 function in bone remodeling systems implicates SOCS3 as a key signaling molecule in bone cell-mediated inflammatory responses. Both pro- and anti-inflammatory functions of SOCS3 have been demonstrated in different types of bone cells. This review provides an overview of the important role of SOCS3 in inflammatory responses of various bone cells and in bone inflammatory disorders such as periodontal disease and arthritis. Understanding the roles of SOCS3 in inflammatory diseases of bone and joints such as arthritis, osteomyelitis, and periodontal diseases is critical to revealing insights into signaling pathways that can be manipulated in potential therapeutic approaches.

## Introduction

Suppressor of Cytokine Signaling 3 (SOCS3) functions as a negative regulator of cytokine signaling by inhibiting the Janus Kinase (JAK)-signal transducer and activator of transcription (STAT) pathways. Expressed ubiquitously in a number of cell types, including macrophages, neutrophils, and T cells, endogenous SOCS3 may have profound actions in the regulation of immunity and inflammation (1, 2). In addition, recent studies have shown SOCS3 to be a significant player in bone-associated inflammatory responses (3–5). However, the underlying mechanisms and signaling pathways regulating SOCS3 expression in osteoblasts, osteoclasts, and other types of bone cells are still not well understood.

There are currently eight known SOCS proteins (SOCS1–7 and CIS) that consist of a variable N-terminal region, a central Src homology-2 (SH2) domain, and C-terminal SOCS box (6). The SH2 domain is responsible for recognizing phosphotyrosine motifs of substrates such as JAK or activated cytokine receptors (7). SOCS proteins bind to activated cytokine receptors or their associated JAKs through the SH2 domains, thereby regulating signal transduction (7). The SOCS box domain constitutes an approximately 40-amino acid motif that binds elongins B and C as well as Cullin 5 and RING finger protein Rbx2 to catalyze the ubiquitination of bound signaling proteins (7, 8). The C-terminal box is thought to regulate SOCS3 protein stability by interacting with elongin C (9). SOCS1 and SOCS3, the two most potent suppressors of cytokine signaling contain the kinase inhibitory region (KIR) upstream of their SH2 domain that allows for direct inhibition of JAK catalytic activity, which in turn suppresses signaling (8, 10, 11). SOCS3 was initially reported to inhibit cytokine signaling by binding to the activation loop of JAKs; however, it has been shown that SOCS3 also functions by binding directly to specific phosphotyrosine motifs of activated cytokine receptor subunits (9).

## SOCS3 in Osteoblasts

Little data is available for the expression and function of SOCS3 in osteoblasts. Recently, several studies have established the up-regulation and suppression of SOCS3 in osteoblast-like cell lines and primary osteoblasts by multiple stimulators (3–5). Additionally, a negative regulatory role of SOCS3 following pro-inflammatory stimuli has been demonstrated in osteoblasts (3–5, 10). It was shown that growth hormone (GH) induces the expression of SOCS3 in UMR 106 cells, a rat osteosarcoma line that exhibits osteoblast-like properties and expresses a GH-response JAK2/STAT5 signaling system (3). Furthermore, pre-treatment of UMR 106 cells with 1, 25-(OH_2_D_3_) delayed and decreased the GH-induced SOCS3 protein expression (3). This in turn suggests the possibility that 1,25-(OH_2_D_3_)-mediated down-regulation of SOCS3 regulates GH signaling in UMR 106 cells.

To determine the function of SOCS3 *in vitro*, an osteoblast-like cell line, MC3T3-E1, and primary osteoblasts were stimulated with lipopolysaccharide (LPS) from *Escherichia coli (E. coli)*. Cellular RNA was collected to evaluate gene expression of interleukin (IL)-6 and matrix metalloproteinase (MMP)-13, two notable pro-inflammatory molecules associated with bone diseases. It was shown that LPS induces both IL-6 and MMP-13 gene expression in osteoblasts (4, 5). Additionally, LPS transiently induced SOCS3 in osteoblast-like cells, thereby contributing to the early stage inhibition of LPS-induced IL-6 expression (4). However, endogenous levels of SOCS3 decreased continuously following LPS stimulation after 6 h. Moreover, overexpression of SOCS3 drastically inhibited production of LPS-induced IL-6 by inhibiting transcription factor, CCAAT/enhancer-binding protein (C/EBP)β, DNA binding activity (4). This finding suggests SOCS3 is an important endogenous inhibitory regulator of signal transduction in osteoblasts following LPS stimulation. However, the study did not report a direct interaction between SOCS3 and C/EBPβ. Nevertheless, the data supports that C/EBPβ is critical for LPS-induced IL-6 expression in osteoblasts (4).

SOCS3 exhibited similar inhibitory actions on MMP-13 gene expression in osteoblast-like MC3T3-E1 cells and primary murine calvarial osteoblasts (5). We have recently demonstrated that through overexpression and knockdown of SOCS3 protein, SOCS3 suppresses MMP-13 transcriptional activity following LPS stimulation by regulating the p38 MAP Kinase signaling pathway (5). This observation is consistent with a previous study’s results that showed that LPS-stimulated MMP-13 transcriptional activation was significantly reduced by inhibition of p38 MAPK in murine periodontal ligament (PDL) fibroblasts (12).

Taken together, these findings establish an elementary understanding of the expression and function of SOCS3 in osteoblasts. It is possible that SOCS3 down-regulates additional pro-inflammatory mediators induced by LPS in osteoblasts, thereby playing a critical role in osteoblast-mediated immune signaling. In addition, molecular details of SOCS3 regulation of signal transduction pathways downstream to Toll-like-receptor (TLR) 4 binding remain to be determined.

## SOCS3 in Osteoclasts

The osteoclast is a bone-resorbing cell that, in conjunction with the osteoblast, contributes to the maintenance of healthy bone metabolism. Excess osteoclast activity leads to the excessive bone loss seen in common bone diseases such as osteoporosis, periodontitis, and other clinical conditions (13). While several studies have established the expression of SOCS3 in osteoclasts, the functional activity of the protein remains controversial, as studies have reported SOCS3 to be both an inducer and inhibitory regulator of cytokine signaling (13–15).

The expression of SOCS3 in osteoclasts is induced by a number of factors, the most studied being transforming-growth factor β (TGF-β) and receptor of activator of NF-κB ligand (RANKL) (13, 14). Specifically, SOCS3 gene expression is primarily induced by the STAT family of transcription factors; however, studies have suggested that SOCS gene expression can also be achieved via JAK/STAT-independent activation. For example, in osteoclast progenitors, RANKL signaling was mediated by the activation of NF-κB and AP-1 following LPS and IL-1β stimulation; the promoter region of the SOCS3 gene also contains NF-κB and AP-1 binding sites as well as STAT-response elements, suggesting that its expression can be induced at transcriptional level by these stimuli (14). Furthermore, sRANKL treatment increased mRNA levels of both SOCS1 and SOCS3 in bone marrow-derived monocytes/macrophages (M/MO) cells as osteoclast progenitors in a time-dependent manner (14). In fact, an increase in SOCS levels was observed as early as 1 h after the addition of sRANKL, peaking at 5 h, and continuing until 22 h, although levels decreased after 8 h (14). In addition, sRANKL treatment caused direct induction, and did not increase levels of SOCS with the addition of cycloheximide (14). Interestingly, a recent study reported that while tacrolimus, a macrolide immunosuppressant that chiefly interferes with T cell activation, was found to decrease RANKL expression in an arthritis mice model compared to control mice, tacrolimus significantly induced SOCS3 mRNA expression in the arthritis model (47).

Another well-studied inducer of SOCS3 gene expression is TGF-β (13, 15). It was shown that TGF-β induces a rapid and continuous increase in SOCS3 mRNA expression in non-committed precursors and mature osteoclasts (13). Moreover, the combined treatment of TGF-β and RANKL led to a sustained increase in SOCS3 mRNA levels that were about seven times control levels (13). SOCS3 expression is also induced by IL-3, IL-4, IL-13, GM-CSF, IFN-β, and IFN-γ in a number of cells, including bone marrow cells via STAT-dependent transcription (13, 16).

Contradictory roles of SOCS3 in osteoclasts have been reported. One study showed that during periods of TGF-β treatment and low bone resorption, TGF-β stimulates osteoclast differentiation to support survival through transcriptional activation of SOCS3; however, when bone resorption levels are high, TGF-β inhibit both differentiation and mature osteoclast apoptosis (15). A second study reported that overexpression of SOCS3 mRNA in osteoclasts promotes osteoclastogenesis via suppression of IFN-β (13). In contrast, a third study found that non-arthritic SOCS3–/Δvav mice, which lack SOCS3 protein in the hematopoietic and endothelial cell compartment, exhibited increased basal osteoclast expression *in vivo* compared with wild type (WT) mice, suggesting a negative regulatory role for SOCS3 (17). Despite these discrepancies, it has been clearly established that SOCS3 plays a key role in regulation of both inflammation and bone.

## SOCS3 in Chondrocytes

Increased chondrocyte apoptosis is one possible pathologic mechanism responsible for cartilage damage. Several studies have shown that the expression and function of SOCS3 in chondrocytes play critical roles in preventing cartilage loss in conditions such as osteoarthritis (OA) and rheumatoid arthritis (RA) (18–22). Specifically, SOCS3 works by inhibiting the signaling pathways of pro-inflammatory cytokines such as IL-6 via STAT3 phosphorylation suppression, which in turn would no longer inhibit chondrocyte proliferation.

To assess the role of SOCS3 in chondrocytes, rat chondrosarcoma chondrocytes (RCS) were used to model fibroblast growth factor receptor (FGR) three-related skeletal dysplasia (18, 20). When activated, FGRs attenuate cartilage growth. STAT activation is a key feature of the initiation and perpetuation of arthritis in mice (23). Stimulation of RCS cells with FGF 2 which binds FGR3 led to STAT3 phosphorylation, but not STAT1, STAT5, or STAT6 phosphorylation (20). Moreover, protein levels of STAT3 increased as well; however, comparison with mRNA levels indicated that FGF2 might additionally accumulate STAT3 independent of transcription (20). It was shown that FGF2 also increased levels of STAT3 protein in mouse limb explant cultures (20). When RCS chondrocytes were treated with IFN-γ, IL-6, IL-11, and leukemia inhibitory factor (LIF), STAT3 was activated. Interestingly, however, the combined treatment of the cytokines and FGF2 impaired STAT activation (20). In addition to IL-1, FGF2 has also been shown to induce SOCS3 in both RCS chondrocytes (20) and in primary cultured articular chondrocytes (18). Since FGF2 inhibits cytokine (IL-6 and IFN-γ)-mediated activation of STAT3 in RCS cells and also up-regulates SOCS3 protein levels, it is possible that the inhibition of STAT3 is due to the increased presence of SOCS3, which regulates STAT3 in a negative feedback loop. However, whether the expression of functionally active SOCS3 negatively regulates IL-6 signaling in chondrocytes has yet to be investigated.

When SV40 large T antigen-immortalized H4 chondrocytes derived from hip articular cartilage of C57BL/6 mice were treated for 2 h with IL-1, a cytokine whose levels are elevated in arthritic synovial fluid (19), phosphorylation of STAT3 was induced. Importantly, RT-PCR revealed that IL-1 stimulation increased levels of SOCS3 mRNA in chondrocytes by about 700% compared with control levels, and Western blot analysis showed a prominent increase in SOCS3 protein as well (19). Direct stimulation of chondrocytes with IL-1-induced high SOCS3 protein levels both *in vivo* and *in vitro* (19). Furthermore, forced expression of SOCS3 in H4 chondrocytes via transduction inhibited phosphorylation of STAT3, indicating antagonistic activity of SOCS3 (19).

Recently, studies have also shown the increased expression of SOCS3 in human arthritic chondrocytes compared with control chondrocytes (22, 24). For example, there was greater expression of SOCS3 mRNA in chondrocytes obtained from the cartilage of patients with OA and RA than in chondrocytes from the cartilage of patients with femoral neck fracture (22). Furthermore, a significant positive correlation between increased SOCS3 expression and MMP-13 expression was observed (22). These findings demonstrate the important function of SOCS3 protein in human cartilage pathology. Whether SOCS3 plays a protective role in cartilage through anti-inflammatory pathways or functions as a mediator for destructive mediators such as MMP-13 remains to be determined.

## SOCS3 in Synoviocytes

An important role for SOCS3 is the suppression of signal transduction pathways that lead to excessive inflammation and bone loss. Inhibition of STAT and JAK is considered a critical therapeutic aim in order to prevent bone destruction in RA (25–27). On the other hand, SOCS3 is closely related to osteoclastogenesis, thus making it a prime candidate of interest to study in bone diseases. Synovial fibroblasts (SFs) are cells that play critical roles in the pathogenesis of chronic inflammatory diseases; particularly, SFs participate in cartilage destruction and producing pro-survival cytokines, chemokines, and angiogenic factors (28). Many of these cytokines, such as IL-6, activate STAT3, which in turn suppresses pro-apoptotic pathways. One study demonstrated that STAT3 is important for RA synoviocyte survival (28). Interestingly, Han et al. shows that the inhibition of p38 MAP kinase suppresses the production of IL-1β-induced cytokines and MMPs in a human fibroblast-like synoviocyte cell line, MH7A, while it induces the expression of SOCS3 and interferon regulatory factor 1 (IRF-1) (29). In another recent study, Mori et al. shows that IL-1β and TNF-α-initiated IL-6-STAT3 activation plays a critical role in mediating the expression of inflammatory cytokines and receptor of nuclear factor kappa B ligand (RANKL) in murine osteoblastic and fibroblastic cells (27). Importantly, they demonstrate that the drug CP690, 550, or Tofacitnib, a Jak3 inhibitor, significantly inhibits collagen-induced arthritis (CIA) through reducing the expression of IL-6 family cytokines and RANKL, suppressing both inflammation and joint destruction (27). These results together with their previous reports that overexpression of SOCS3 or dominant negative STAT3 in joints effectively ameliorated CIA models (30) indicate that IL-6–STAT3–mediated cytokine amplification loop plays a key role in promoting sustained inflammation and joint destruction during arthritis, and this loop can be negatively regulated by SOCS3.

Tacrolimus was shown to exhibit anti-arthritic actions by regulating inflammatory cytokine production in RA (31, 32). Additionally, tacrolimus prevents differentiation of cells into mature osteoclasts, thereby conferring protection against bone resorption (33, 34). One recent study reported that tacrolimus significantly increased SOCS3 mRNA levels in affected joints in an arthritis mouse model induced by K/BxN serum when compared to non-treated arthritic mice (35). Furthermore, treatment with tacrolimus in addition to IL-6/sIL-6R stimulation significantly decreased receptor activator of NF-κB ligand (RANKL) expression as well as JAK2 and STAT3 phosphorylation in fibroblast-like synoviocytes (35). Western blot analysis revealed that tacrolimus also increased SOCS3 protein expression in a dose-dependent manner (35). Interestingly, while down-regulation of JAK-STAT activation induced the expression of SOCS3, the expression of SOCS1, and CIS1 did not change (35). Since fibroblast-like synoviocytes constitute a potent source of RANKL production in patients with RA, the blockade of RANKL expression by the SOCS3 pathway in fibroblast-like synoviocytes may be important in the regulation of osteoclast differentiation for bone erosion in RA. Together, these data suggest that the JAK2-STAT3-SOCS3 signaling pathway in fibroblast-like synoviocytes may play a critical role in the pathogenesis of RA.

## SOCS3 in Periodontal Diseases

*Porphyromonas gingivalis* (*P. gingivalis*) is a gram-negative bacterium involved in the pathogenesis of periodontal disease. *P. gingivalis* regulates pathways that control cytokine signaling; for example, *P. gingivalis* infection of epithelial cells can stimulate the production of pro-inflammatory cytokines such as IL-1β and suppress anti-inflammatory cytokines such as IL-10 (36). *P. gingivalis* also modulates cell apoptosis via JAK signal transduction as well as STAT and Akt pathways (36).

The human genome consists of many miRNAs, which are known to be key players in pathway network control of cellular processes such as inflammation and apoptosis (36). One recent study showed that *P. gingivalis* significantly induces the expression of 14 miRNAs in primary cultures of gingival epithelial cells (36). Among them, miR-203 has defined targets, and has been implicated to regulate cytokine signaling. Thus, targeting miR-203 in periodontitis may have important implications in therapy, as miR-203 expression has been reported in RA and psoriatic arthritis (36). One known cytokine downstream of miR-203 is SOCS3 (36). It was shown that *P. gingivalis* infection reduced levels of SOCS3 mRNA as it induced miR-203 and that regulation of SOCS3 by *P. gingivalis* was dependent on miR-203 in gingival epithelial cells (36). Furthermore, the decrease of SOCS3 increased activation of STAT3 (36). Interestingly, in an unpublished study, we determined that LPS from the *P. gingivalis* pathogen significantly increases levels of SOCS3 in THP-1 cells, a human monocytic cell line. However, we also showed that *P. gingivalis* LPS stimulation of primary calvarial osteoblasts did not change SOCS3 mRNA levels compared to control cells.

To determine the relationship between inflammatory cytokines potentially induced by *P. gingivalis* and SOCS3, human PDL cells were stimulated with pro-inflammatory cytokines such as IL-1β and IL-6 (37). RT-PCR and Western blot analysis revealed that IL-1β and IL-6 both induced SOCS3 expression in PDL cells. Moreover, overexpression of SOCS3 suppressed secretion of IL-8 and monocyte chemoattractant protein (MCP)-1 in PDL cells by inhibiting phosphorylation in downstream signaling (37). Taken together, these results suggest that the induction of pro-inflammatory cytokines by *P. gingivalis* can lead to SOCS3 regulation of destructive cytokine signaling, thus potentially preventing excessive inflammation and bone loss seen in chronic periodontal diseases. Furthermore, these data suggest that a failure of SOCS3 expression of function plays a role in osteolytic periodontal lesions. However, this hypothesis needs to be determined by *in vivo* animal models.

## SOCS3 and Inflammatory Arthritis

A key characteristic feature of any form of arthritis is bone loss. Bone and cartilage destruction result from overwhelming osteoclast activity compared with osteoblast activity and from prolonged inflammation. These conditions can arise from excessive production of pro-inflammatory cytokines. For example, in OA, articular chondrocytes induce the production of IL-1β and TNF-α (21).

It was reported that STAT3 levels correlated positively with inflammation severity (38). Interestingly, immunohistochemistry comparison of STAT3 activation in RA and OA patients with anti-phospho-STAT3-specific antibodies showed that STAT3 was activated in the nucleus of RA synovial cells but not in that of OA cells (30). Furthermore, Northern blot analysis revealed that cytokine signal suppressor (CIS3) was strongly expressed in RA synovial tissues from RA patients, but only scarcely in OA synovial tissues (30). Since SOCS3 inhibits STAT3 signaling in a number of cells, it is not surprising that SOCS3 was also shown to negatively regulate innate and adaptive immunity in both antigen-induced arthritis (AIA) and CIA (30).

Recent studies demonstrate that T helper 17 (T_h_17) cells play a critical role in the development of arthritis (39, 40). Interestingly, Mori et al. suggest that STAT3 pathway contributes to RA by inducing T_h_17 cell development (27). Importantly, SOCS3 is a major regulator of IL-23-mediated STAT3 phosphorylation and T_h_17 generation (41). Furthermore, TGF-β promotes T_h_17 cell development via inhibition of SOCS3 (42). These studies suggest that SOCS3 may regulate the development of arthritis through T_h_17 differentiation.

To determine the function of endogenous SOCS3 in arthritis, one study compared the severity of mBSA/IL-1-induced inflammatory arthritis in WT, *Socs3*^+^*^/^*^Δ^*^vav^*, and *Socs^−/^*^Δ^*^vav^* mice (17). *Socs3*^+^*^/^*^Δ^*^vav^* mice were used as controls. *Socs^−/^*^Δ^*^vav^* mice underwent deletion of SOCS3 in all hematopoietic and endothelial cells (17). The *Socs^−/^*^Δ^*^vav^* mice exhibited characteristics of exacerbated inflammatory arthritis, such as dramatic synovial hyperplasia, intense pannus erosion into the bone with increased inflammatory exudate, reduced basal trabecular bone volume, and lack of synovial granulomas (17). Furthermore, cartilage destruction and proteoglycan loss was significantly increased in SOCS3 deficient mice. *Socs^−/^*^Δ^*^vav^* mice also displayed elevated numbers of osteoclasts, which correlates with the bone loss seen in arthritis (17). This observation suggests that endogenous SOCS3 inhibits basal osteoclastogenesis and that in non-arthritic *Socs^−/^*^Δ^*^vav^* mice, osteoblasts express SOCS3 to compensate for bone loss resulting from increased osteoclast activity. Macrophages are important for arthritis (43). Specifically, SOCS3 inhibits M1 macrophage polarization and inflammation (44). These findings together suggest that SOCS3 is an indispensable negative regulator of inflammation in arthritis (17, 30).

## Conclusion

**Figure 1:**
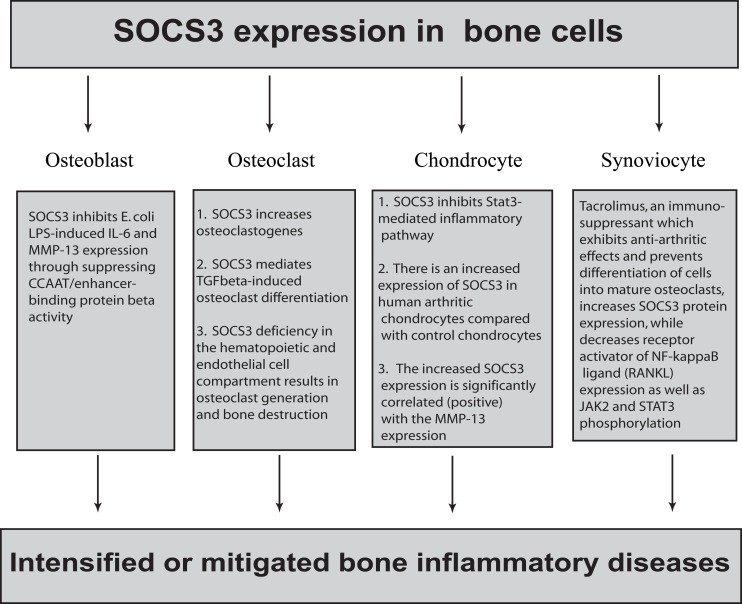
**The complex role of SOCS3 in bone cells**. SOCS3 is expressed in osteoblasts, osteoclasts, chondrocytes, and synoviocytes. In osteoblasts, SOCS3 inhibits *E. coli* LPS-induced IL-6 and MMP-13 expression by suppressing CCAAT/enhancer-binding protein β activity. In contrast, SOCS3 positively regulate osteoclastogenesis by blocking the inhibitory effect of inflammatory cytokines on receptor activator of the NF-kappaB ligand-mediated osteoclast differentiation signals. SOCS3 deficiency in the hematopoietic and endothelial cell compartment results in osteoclast generation and bone destruction. In chondrocytes, SOCS3 inhibits the Stat3-mediated inflammatory pathway. In contrast, there is an increased expression of SOCS3 in human arthritic chondrocytes compared with control chondrocytes. There is a strong positive correlation between SOCS3 expression and MMP-13 suppression. Tacrolimus, an immunosuppressant, was shown to exhibit anti-arthritic effects by regulating inflammatory cytokine production in RA. Additionally, tacrolimus prevents differentiation of cells into mature osteoclasts, thereby conferring protection against bone resorption. In fibroblast-like synoviocytes, tacrolimus increases SOCS3 protein expression while it decreases receptor activator of NF-κB ligand (RANKL) expression as well as JAK2 and STAT3 phosphorylation.

In the past decade, the expression and function of SOCS3 have been intensively studied in immune cells such as macrophages and T cells. Most of these studies are focused on the regulation loop of cytokine-STAT/JAK-SOCS3. In addition, SOCS3 expression in these cells can also be induced by a variety of other inflammatory stimuli including bacterial products (45). However, detailed mechanisms by which SOCS3 regulates signaling pathways distinct from STAT/JAK remain incomplete and controversial. Interestingly, recent studies indicate that SOCS3 is also expressed in many other cell types including osteoblasts, osteoclasts, chondrocytes, and synoviocytes (Figure 1). However, the expression of SOCS3 in these cells in response to various inflammatory stimuli and its function in bone inflammatory diseases are still largely unknown. In addition, SOCS3 seems to have opposing functions in regulating inflammatory responses in these cells (Figure 1). For example, while SOCS3 inhibits LPS-induced IL-6 and MMP-13 expression in osteoblasts (4, 5), SOCS3 as well as SOCS1 positively regulate osteoclastogenesis by blocking the inhibitory actions of inflammatory cytokines on receptor activator of the NF-kappaB ligand-mediated osteoclast differentiation signals (46). Thus, the role of SOCS3 during bone inflammation is complex; more details of the SOCS3 pathway are necessary for a better understanding of the mechanisms of various bone inflammatory diseases.

In addition, the net effect of SOCS3 may be dependent on when and in which cell type SOCS3 plays a major role. In this regard, use of inducible SOCS3 knock-out mice in different bone cells may be a realistic future direction. Although the functional investigation of SOCS3 in the bone inflammatory response is still a challenge, these endeavors will be fruitful. Elucidating SOCS3 function in various bone cells will identify prospective targets for a new generation of drugs that target inflammation-associated bone diseases. Moreover, identification of SOCS3 interacting proteins will lead to a better understanding how SOCS3 regulates bone inflammatory responses through transcriptional and/or post-transcriptional control of downstream genes such as IL-6 and MMP-13.

## Conflict of Interest Statement

The authors declare that the research was conducted in the absence of any commercial or financial relationships that could be construed as a potential conflict of interest.
